# COMET (Composite Outcomes of Mesh vs suture Techniques for prolapse repair)- Protocol for a single blind randomized controlled multicenter trial testing surgical innovation in female pelvic surgery

**DOI:** 10.1371/journal.pone.0308926

**Published:** 2024-10-24

**Authors:** Lina Roa, Maryse Larouche, Momoe Hyakutake, Erin A. Brennand, Ola Malabarey, Nicole Koenig, Terry Lee, Joel Singer, Wei Zhang, Lori A. Brotto, Roxana Geoffrion

**Affiliations:** 1 Department of Obstetrics & Gynecology, University of British Columbia, Vancouver, Canada; 2 St Mary’s Research Centre, Montreal, Quebec, Canada; 3 Department of Obstetrics and Gynecology, McGill University, Montreal, Quebec, Canada; 4 Department of Obstetrics & Gynecology, University of Alberta, Edmonton, Canada; 5 Departments of Obstetrics & Gynecology and Community Health Sciences, Cumming School of Medicine, University of Calgary, Calgary, Canada; 6 Department of Obstetrics & Gynecology, Division of Female Pelvic Medicine & Reconstructive Surgery, McMaster University, Hamilton, Canada; 7 Centre for Advancing Health Outcomes, University of British Columbia, Vancouver, Canada; 8 Faculty of Pharmaceutical Sciences, University of British Columbia, Vancouver, Canada; University of Bremen: Universitat Bremen, GERMANY

## Abstract

**Background:**

Pelvic organ prolapse (POP) increases in incidence and severity with aging. At least 1 in 4 women seek pelvic floor care and many more suffer with concurrent symptoms of bowel, bladder and sexual dysfunction, which can have a large impact on quality of life. It is estimated that 1 in 5 women will undergo surgery for POP. POP is difficult to cure with existing surgeries and therefore treatment failure and reoperations are common. Surgical innovation in this area is urgently needed and we have developed a novel technique of bilateral sacrospinous vaginal vault fixation with synthetic mesh arms (BSSVF-M). Based on preliminary studies it may be more successful, durable and cost-effective than standard sacrospinous ligament suspension with sutures (SSLS). Preliminary development and exploration studies showed safety and efficacy of BSSVF-M. Following an established framework for research in surgical innovations, we now wish to conduct a randomized comparative effectiveness trial for assessment of this novel technique.

**Methods:**

This is a multi-center randomized controlled trial in Canada comparing the surgical techniques of BSSVF-M vs. SSLS to address apical prolapse. In total, 358 women with symptomatic POP at five centers will be randomized with 80% power to detect a 15% difference in primary composite outcome and accounting for a 15% loss to follow-up over 2 years. The primary objective is to investigate BSSVF-M vs. SSLS using an established composite of 3 objective signs and 1 subjective symptom of POP measured 2 years postoperatively. Secondary objectives: 1) To determine changes in condition-specific pelvic symptoms, quality of life, pain and condition-specific body image post BSSVF-M vs. SSLS using validated questionnaires; 2) To determine changes in sexuality post BSSVF-M vs. SSLS; 3) To determine global impression of improvement, adverse events (validated classification scheme), reoperations and health utility post BSSVF-M vs. SSLS; 4) To determine the cost-effectiveness of BSSVF-M vs SSLS. Study Registration at clinicaltrials.gov (NCT02965313).

**Discussion:**

There is a need for innovation to improve the surgical approach to vaginal apical suspension. Despite controversies with mesh, it has been shown to be safe when used appropriately and to have higher durability when compared with sutures. As well, the importance of restoring anatomy and tension-free surgical approach in pelvic reconstructive surgery has led to better long-term outcomes and fewer side effects. These principles have been applied when developing the novel BSSVF-M technique. Anticipated challenges of this trial include recruitment, compliance problems and loss to follow up However, the robust methodology will provide evidence on the best surgical approach to correct POP, a common condition among aging women.

## Background

The pelvic floor’s muscles, nerves, ligaments, and fascial network create a dynamic elastic support for intraabdominal organs. Pelvic organ prolapse (POP) occurs when the capacity to accommodate pressure and stretch is exceeded, and increases with childbirth and aging [1] POP negatively affects the quality of life of aging women. Approximately half of parous women will have symptoms and one in five women will require reconstructive surgery [2]. Vaginal apical suspension is a standard approach to surgical reconstructive treatment. The sacrospinous ligament (SSL) is a strong, connective structure lying between the ischial spine and the lateral edge of the sacrum. The SSL has commonly been used to repair the apical compartment prolapse [3]. The most common standard vaginal apical repair technique is the sacrospinous ligament suspension with sutures (SSLS). Overall, vaginal suspension techniques for apical prolapse showed higher risk of apical anatomic recurrence compared with abdominal sacrocolpopexy with synthetic mesh [4]. Moreover, SSLS in particular has been associated with a higher risk of transient buttock pain when compared with other vaginal surgeries such as uterosacral ligament suspension (USLS) [4]. Therefore, surgical innovation is desirable to mitigate some of the limitations of currently available surgical techniques for vaginal vault prolapse.

This research investigates a variation of the standard SSLS: bilateral sacrospinous vaginal vault fixation with synthetic mesh arms (BSSVF-M), which may be more successful, durable and cost-effective than SSLS. This novel and versatile surgical technique uses two synthetic polypropylene mesh arms to suspend the vaginal apex (with or without a uterus in place) to the sacrospinous ligaments bilaterally. The bilateral sacrospinous vaginal vault fixation with synthetic mesh arms (BSSVF-M) restores uterosacral support, creating an anatomically correct midline configuration of the vaginal axis with minimal tension [5]. The tension-free aspect of the mesh in BSSVF-M is a key difference with SSLS, which pulls the vagina tight against ligaments, potentially causing buttock pain or suture detachment with potential return of POP after SSLS. The size of the mesh can be customized to individual anatomic variations of a shorter vaginal canal (longer mesh arms) or a shallow/narrow pelvis with ischial spines closer to the surface (shorter mesh arms). A magnetic resonance imaging study demonstrated that BSSVF-M restores optimal support to the pelvis [5]. An exploratory prospective cohort study, showed BSSVF-M to be safe, easily taught to other surgeons and successful in 77% of women at one year which is superior to the reported 60% success of the SSLS [6]. Another study using a different technique of sacrospinous ligament fixation with mesh has also reported on its effectiveness and safety at one year follow up [7]

A Cochrane review on surgical management of POP concluded that *“adequately powered RCTs with blinding of assessors are urgently needed […] they particularly need to include women’s perceptions*.*”* [8]. At this stage, a comparative RCT of BSSVF-M and SSLS, evaluating composite success, is warranted before widespread adoption of this novel technique.

**Fig 1:**
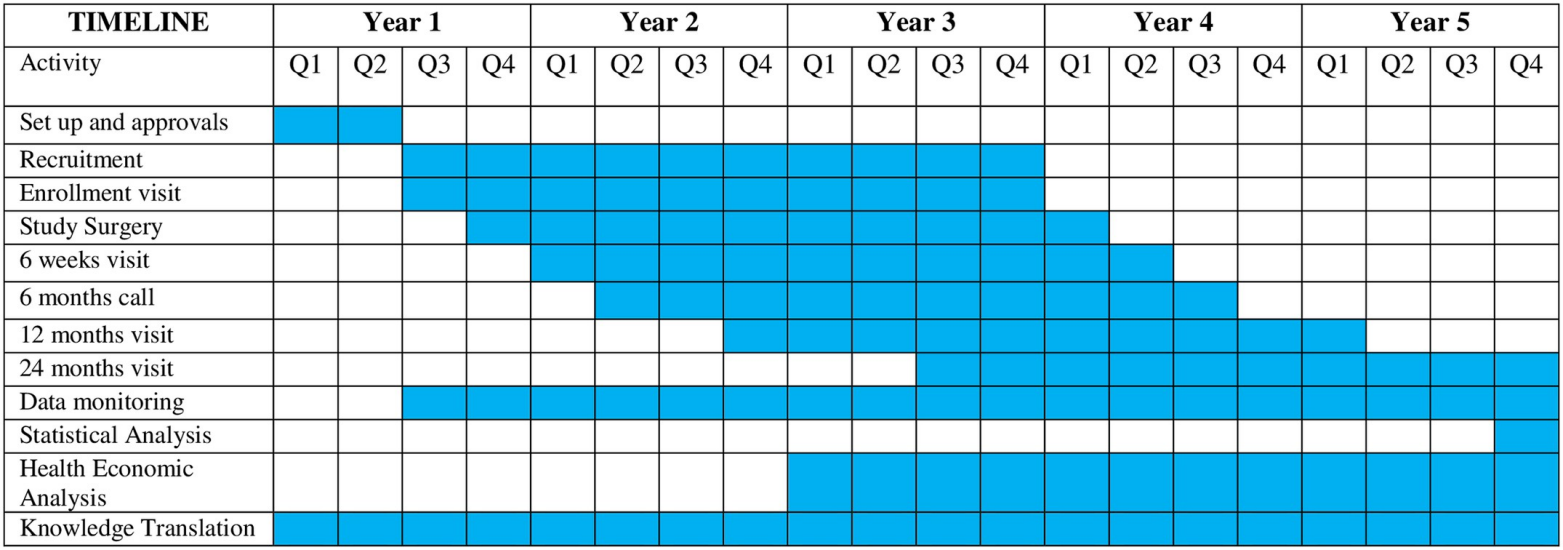
Schedule of enrollment, interventions, and assessments.

The primary objective of this randomized controlled trial is to compare BSSVF-M *vs*. SSLS via a composite subjective and objective outcome measure at 2 years. The secondary objectives are to determine condition-specific urinary, bowel and POP symptoms, quality of life, new onset pelvic pain, condition-specific body image, sexuality, global improvement, adverse events, reoperations, health utility and healthcare utilization and costs up to 2 years post BSSVF-M *vs*. SSLS. Validated questionnaires and adverse event schemes will be used. A Markov model will estimate 10-year health benefits and costs of surgery. Our protocol follows evidence-based recommendations for clinical protocols (Fig 1).

## Materials and methods

### Study setting

The study will take place in five Canadian tertiary care urogynecology centers in Vancouver, Edmonton, Calgary, Montreal and Hamilton.

### Eligibility

#### Inclusion criteria

- Females over 19 years of age
- Able to read and write in English or French, or alternatively have someone to translate for them
- Able to follow up with clinic visits for up to two years after surgery
- Diagnosed with bothersome pelvic organ prolapse
- Patients with concurrent anterior or posterior wall prolapse requiring an anterior or posterior repair will be included.

#### Exclusion criteria

- Women who wish uterine preservation
- Prior pelvic radiation
- Prior vaginal mesh surgery for prolapse
- Prior vaginal mesh exposure
- Presence of vaginal pain from pelvic floor muscle spasm documented at baseline visit
- Plan for synthetic vaginal mesh insertion for prolapse, at the same time, at locations other than the top of the vagina (concurrent need for synthetic anti-incontinence sling is NOT an exclusion)
- Immune compromise; chronic steroid use
- Current smoker
- Currently pregnant or breastfeeding
- Presence of pain syndromes likely to cause a heightened sensitivity to pelvic pain (example: fibromyalgia or painful bladder syndrome)

### Recruitment

Patients will be recruited once they have decided to proceed with surgical management for apical prolapse. In our practice, surgical management is offered after patients have failed conservative therapies. Trained research personnel will introduce the trial to patients who will be shown educational materials regarding pelvic organ prolapse and the trial interventions. Patients will have an informed discussion with the participating personnel who will obtain written consent for participation. Patients will receive information sheets and a copy of the consent form. Recruitment will likely span over 3–5 years.

Based on prior experience with the pilot study of BSSVF-M and OPTIMAL, we anticipate approximately 90% of invited women will volunteer and 5% of those will change their mind about participation at baseline [6]. All surgeons are experts at vaginal surgery for POP (including SSLS). They do not currently offer BSSVF-M outside research studies. According to national credentialing organizations [9] and our surgical survey, surgeons require at least 5 and on average 8 procedures to become familiar with a new vaginal POP technique. Assistance with 8 cases will be provided to the surgeon untrained to perform BSSVF-M. In addition, video instruction will be provided to standardize procedure and materials.

Participating sites and starting date for recruitment include the University of British Columbia (May 2017), University of Alberta (June 2017), McGill University (September 2017), MacMaster University (June 2019) and University of Calgary (February 2021). Our study was significantly delayed due to the Covid global pandemic at different time periods at multiple sites (each site having different set of delapys as per the different provincial mandates). Across Canada there were mandatory interruptions in clinical research activities and research personnel were not allowed to enter the hospital for recruitment purposes. Furthermore, elective surgeries were cancelled at most sites, prolonging waiting times for surgery. At the current rate of recruitment, we estimate we will conclude recruitment by 2025.

### Randomization and blinding

Random allocation is via a password-protected web-based allocation system. A statistician will generate a list of random allocations using SAS PLAN, stratified by surgeon, and using randomly sized permuted blocks to prevent bias in guessing the next treatment allocation. Although all surgeons will be skilled in both techniques, differences in experience may impact outcomes. By stratifying for surgeon, we ensure similar numbers of allocations to each treatment in each hospital. Patients will be enrolled by research personnel at each participating centers. The site coordinator will randomize the patient prior to surgery using the web-based allocation system. Patients will be blinded to the type of procedure received until 2 years after surgery. Research personnel evaluating outcomes at each in person visit will be blinded to the type of procedure received by participants.

In the event of complications arising from either surgery, patients or assessors may become aware of the group assignment. For example, if a patient develops exposure of the vaginal mesh, the pelvic examination will reveal this at various time points and surgery may be needed to remove the mesh exposed in the vagina. This may introduce bias in assessments at various time points, however it will likely not affect the primary composite outcome measure at 2 years. Based on our prior experience with BSSVF-M, we anticipate unblinding complications to be less than 5% of the total sample. The primary surgeon who deals with complications and need for reoperation is not involved in pelvic examination assessments. Most complications will be corrected by 2 years, so the blinded assessor at 2 years will be unable to identify group assignment.

### Interventions

Patients with symptomatic POP will be randomized to BSSVF-M or SSLS. Both surgeries are performed through the same vaginal incisions. BSSVF-M uses bilateral synthetic polypropylene mesh arms for support of the vaginal wall, the mesh arm’s length and width are tailored to each patient’s pelvic dimensions intra-operatively to minimize tension and amount of mesh material used [10]. SSLS uses two prolene sutures attached uni- or bilaterally [3, 8]. Group assignment will remain blinded through trial end. Surgeons will receive group allocation immediately prior to surgery. To diminish bias, research personnel conducting pelvic exams to evaluate the primary outcome will be blind to the type of procedure.

### Outcome measures

#### Primary, at 2 yrs

Composite outcome of 3 objective signs and 1 subjective symptom of POP (yes/no answers). The definition of surgical success, used in the OPTIMAL trial [11] is the absence of all of the following: (1) objectively recorded, via POP quantification (POP-Q) [12], recurrent POP of the top of the vagina beyond the upper third of the vaginal canal; (2) objectively recorded, via POPQ, recurrent POP of the anterior or posterior vaginal walls beyond the hymenal ring (vaginal entrance); (3) vaginal bulge symptoms reported by the patient, as indicated by an affirmative response to *“Do you usually have a bulge or something falling out that you can see or feel in the vaginal area*?*”* and any response other than *“not at all”* to the question *“How much does that bother you*?*”* (4) re-treatment for prolapse by either surgery or pessary (conservative treatment with insertion of a silicone ring in the vagina for support). The POPQ is a validated quantification system with adequate interrater reliability which measures, in centimeters, the extent of vaginal wall descent with respect to a reference point at the hymenal ring [12]

#### Secondary, at 6 weeks, 6, 12 and 24 months

Change in maximal POP of vaginal compartments via POPQ, obtained by research personnel (nurse or clinical fellow) trained in POPQ and blinded to group allocation. POP will be quantified and compared to baseline. Numerical questionnaire scores will be compared between the two groups from baseline to 6 weeks, 12 and 24 months postoperatively. Change in questionnaire scores between the 2 groups from baseline to 6 weeks, 12 and 24 months postoperatively: Pelvic Floor Distress Inventory (PFDI-20) and the Pelvic Floor Impact Questionnaire (PFIQ-7) [13]; the short form McGill pain questionnaire [14]; a validated gender-specific body image scale specifically for POP [15].Change in questionnaire scores between the 2 groups from baseline to 12 and 24 months: Pelvic organ prolapse/ Incontinence Sexual Questionnaire, IUGA-Revised (PISQ-IR)^19^. Difference between global impression of improvement (Likert Scale) of the 2 groups at 6 weeks, 12 and 24 months postoperatively. Difference between the 2 groups in length of surgery, postoperative hospital stay, complications such as pain or mesh exposure, reoperations and other unexpected adverse events categorized using the Clavien Dindo validated classification system for surgical complications [16]. Health utilities will be measured via the EuroQol instrument (EQ-5D-5L) [17] and direct healthcare utilization and costs will be collected by the healthcare service utilization questionnaire [18] at baseline, 6 weeks, 6, 12 and 24 months postoperatively.

#### Health economic evaluation

A cost-effectiveness analysis (CEA) of BSSVF-M *vs*. SSLS will be conducted alongside the 2-year trial from the healthcare system perspective. The incremental cost-effectiveness ratio (ICER) during the 2-year study period will be calculated by dividing the difference in 2-year costs by the difference in the Quality-Adjusted Life Years (QALYs). QALYs will be estimated based on the health utilities at each time point of the 2-year duration. Bootstrap method will be used to estimate the 95% confidence interval (CI) of ICER. The cost-effectiveness acceptability curve (CEAC), plotting the probability that BSSVF-M is cost-effective compared to SSLS at different cost-effectiveness thresholds ($/QALY gained), will be used to represent decision uncertainty. In addition, an alternative CEA will be carried out over 10 years after commencement of surgical treatment by a Markov model. Published Markov models [19, 20] will be adapted to simulate transition between different health states postoperatively and to extrapolate within-trial results over 10 years. Specifically, the potential health states include repaired POP without late/post-operative complications, repaired POP with minor late complications, and repaired POP with major late complications requiring revision surgery. All costs and QALYs will be discounted at a rate of 1.5% per year based on the existing guidelines [21]. Different discount rates at 3% and 5% will be used in scenario analyses. Probabilistic analysis with 1000 Monte-Carlo simulations will be employed to assess the impact of model parameters uncertainty and calculate the 95% CI of the ICER. CEAC will be plotted to represent decision uncertainty.

### Sample size

We conducted a survey of 50 surgeons in Western Canada who indicated the smallest clinically relevant difference to change surgical practice would be an absolute change of 14%. The proportion of success in the control SSLS group was 60.5% in the OPTIMAL trial [11] and the success rate with BSSVF-M was 77% in our one-year pilot study [6]. Our pilot study was small and designed to inform a power calculation for an RCT; it did not study long-term results or cost effectiveness. Studies of mesh vs sutures in surgery for urinary incontinence indicate that mesh is more successful, durable and cost-effective than sutures, [22–25] therefore we expect similar results for POP, with sustained durability of mesh over time. Sample size was determined based on a two-sample test of independent proportions (ie., Chi-square test), using two-sided ∞ = 0.05, anticipated successful outcome rates 60 and 75%, power = 80% and yielded 152 patients/group. To account for an estimated 15% loss to follow-up (patients who never had a follow-up visit or who were deemed successful on their last follow-up), similar to the OPTIMAL trial [26], our sample size was increased to 179 per group for a total of 358 participants.

### Statistical analyses

Data will be analyzed according to the patient’s randomized allocation, i.e., the intention to treat principle. Our primary analytic strategy, following the example of the OPTIMAL trial, will include only patients who come for 2-yr follow-up or who were failures on the last follow-up. Patients who do not come to their 2-yr follow-up but who were failures at last follow-up will be counted as failures. The primary analysis of efficacy will use mixed effects logistic regression to compare the proportion of successes in the two treatment groups adjusting for magnitude of POP at baseline, and patient’s number of prior failed POP surgeries as fixed effects, and surgeon as a random effect. The proposed analysis should provide more statistical power than the Chi-square test due to adjustment of surgeons and other prognostic factors. As a sensitivity analysis, the participants excluded from the primary analysis (see Sample Size section) will have their outcomes imputed using the multiple imputation procedure, MI and MIANALYZE, in SAS. Independent variables in the imputation model will include treatment group, primary outcome at earlier time points if available, magnitude of POP at baseline, number of prior failed POP surgeries and surgeon. Given the relatively small number of surgeons, we will treat surgeon as a fixed effect and test for interaction with treatment. Comparison of continuous secondary outcome measures collected longitudinally will be based on mixed effects regression adjusted for baseline score of the measure, magnitude of prolapse at baseline and number of prior failed POP surgeries.

Separate regression model or non-parametric analysis for each time point might be considered instead if the data do not satisfy the assumptions required in mixed effects regression. By the time that half the patients will have reached their final follow-up, we anticipate that all the study procedures will already have been performed, thus, no interim analyses are planned. Subgroup examination will be considered exploratory as differential effects are unexpected.

### Data management

All trial-related information will be stored securely at the coordinating center in Vancouver. All participant-related information will be stored in locked file cabinets in areas with limited access. All records that contain names or other personal identifiers will be stored separately from study records identified by code number. All local databases will be secured with password-protected access systems. Participants’ study information will not be released outside of the study without the written permission of the participant, except as necessary for monitoring by government and regulatory authorities. To ensure confidentiality, data dispersed to trial team members will be blinded of any identifying participant information.

### Ethics and safety of participants

This protocol and the informed consent forms were reviewed and approved by the Institutional Review Boards (IRB) of the University of British Columbia (protocol #H16-02085 approved on April 14th 2023) and at each participating center with respect to scientific content and human subject regulations. The other centers where IRB approval was obtained were Hamilton Integrated Research Ethics Board (protocol #5957 approved on March 1^st^ 2023), University of Alberta Health Research Ethics Board (protocol #68620 approved on February 28^th^ 2023), McGill University Health Centre (protocol #3720173272 approved on April 02 2022). The responsible IRBs will review the protocol at least annually.

The mesh used at all trial centers is Gynecare Gynemesh^™^ PS, nonabsorbable Prolene^™^ soft Type 1, large pore, midweight mesh [27]. This is a licensed device in Canada, and it is the same mesh that is approved and widely used for abdominal procedures such as sacrocolpopexy. A similar mesh material is also used for general surgery procedures such as hernia repairs, and for vaginal urogynecologic procedures such as mid-urethral slings. Vaginal use of Gynemesh^™^ is currently off label. Since this is an investigator-sponsored study that did not involve the device manufacturer and this is a licensed device for use in humans, the trial is no subject to additional Medical Device Regulation.

An independent committee, the data safety and monitoring board (DSMB) will meet bi-annually and within three months of study termination specifically for this trial according to international standards [28] and each collaborator will make safety and progress reports to the IRBs. We will adapt a DSMB charter that has been used by other trials at our institution. The independent DSMB will be comprised of both clinicians and a biostatistician.

## Discussion

The results of this multi-center randomized controlled trial will provide data on short-term, composite medium-term outcomes, and economic impact of BSSVF-M *versus* SSLS as surgical techniques for apical suspension of the vagina. The BSSVF-M procedure is a novel mesh insertion technique that addresses some of the shortcomings of the SSLS by using mesh to increase durability and by restoring the anatomy in a tension-free fashion to reduce recurrence and side effects common in SSLS.

Vaginal mesh insertion has been associated with significant complications, including exposure, pain, and dyspareunia requiring reoperation [9]. Transvaginal mesh kits, such as Prolift and others, had a large surface area of mesh in contact with the vagina and were thus associated with higher risk of complications [29]. This is why the BSSVF-M procedure was designed to use the smallest possible piece of synthetic material with minimal contact with the vaginal underside and no contact with fresh incisions. Transvaginal tapes use mesh, and this is considered a safe and commonly done procedure for stress urinary incontinence, suggesting that the amount of mesh and the location and application of the synthetic material are key for the safety of the procedure. The use of surgical mesh for prolapse was initially largely unregulated. After the Food and Drug Administration (FDA) increased regulation of vaginal mesh products, device companies have withdrawn most commercially available mesh kits and largely abandoned innovation in this area of women’s health [9]. The increased rigor in the regulatory process mandated improved safety and efficacy, clearer guidelines on appropriate use and training of providers and a call to an increase in high quality research on long term outcomes [9, 30]. This trial answers this call for high quality research to study whether the use of a small piece of mesh for prolapse can optimize outcomes and minimize risks, given that the current use of sutures alone has such high prolapse recurrence rates.

In parallel, recommendations for innovation in surgery have been developed to consider the complexity of procedures and the impact of factors such as operator skill, learning curve, quality variation and perception of equipoise [31]. It has been recommended that development and adoption of new procedures follows reliable evidence acquired through a rigorous Innovation, Development, Exploration, Assessment, Long-term study (IDEAL) paradigm [31, 32]. These ensure innovation in surgery is done in a safe, evidence based and efficient manner. Our trial supports assessment and allows us to continue with long-term outcome studies in future research.

The controversy related to mesh placement does not apply to treatment of stress urinary incontinence such as mid-urethral slings or to the abdominal approach to surgical repair of prolapse such as sacocolpopexy as robust studies have shown the risks of mesh complications associated with these procedures are low [9, 33]. Furthermore, for stress urinary incontinence (SUI), studies have shown that in the long term the use of mesh is more durable than the use of sutures alone. The long-term efficacy of SUI treatment was significantly higher in women undergoing a TVT with mesh compared with a Burch procedure with sutures [34]. With a Burch colposuspension, there is a high risk of needing prolapse surgery likely due to the traction on the endopelvic fascia from the sutures in the Burch procedure [34, 35] while the TVT is a tension-free procedures with the support being more precisely on the mid-urethra [36]. As a result, there is a significant lower rate of prolapse surgery in patients after undergoing TVT versus Burch procedure likely due to anatomical changes in the mechanical properties of the pelvis caused by the suture suspension [34]. These findings highlight the increased durability of mesh compared to sutures as well as the importance of restoring anatomy with a tension free approach to minimize side effects and recurrence. We hypothesize that the BSSVF-M will result in better anatomical correction and tension free placement of mesh will result in less buttock pain or suture detachment and recurrence compared with SSLS.

The Operations and Pelvic Muscle Training in the Management of Apical Support Loss (OPTIMAL) trial, a large RCT comparing 2 POP surgeries, established a stringent definition of success using a composite outcome of objectively measured POP, subjective improvement and the need for another treatment for POP recurrence [11]. As described in the Methods, we have followed these criteria to define our composite outcome. The OPTIMAL RCT examined the 2-year and 5-year outcomes associated with USLS versus SSLS. At 5 years, the failure rates were high at 61.5% in the ULS group and 70.3% in the SSLF group, with no significant difference between the groups [37]. Interestingly, despite increased surgical failure rates at 5 years compared with outcomes at 2 years, prolapse symptoms scores remained improved. Quality of life measures including prolapse distress, urinary distress and colorectal distress significantly improved up until 5 years after surgery in both groups. This trial highlights the need for long-term follow-up in surgical trials for prolapsed [37]. Studies assessing only short- and medium-term outcomes may not detect a difference in durability seen only with long term follow up. We hope to follow up patients in this trial beyond 2 years to assess long term outcomes.

Anticipated challenges for this study include recruitment, compliance problems and loss to follow up. Despite evidence that mesh can be safe when used appropriately, we anticipate many patients will still be hesitant to be enrolled in a trial that involves transvaginal mesh. Compliance with follow-up may be an issue due to the follow up for 2 years. Patients will be screened for future compliance at recruitment and the coordinator at each site will follow each patient quarterly over the 2 years, asking for updated contact information at each time point. The framework of loss to follow-up in a surgical trial has been delineated via the OPTIMAL trial. To mitigate the anticipated 15% loss to follow-up, we will reimburse patient parking and traveling costs with a small stipend. Our rigorous trial of an innovative surgery could demonstrate improved subjective and objective success, as well as cost-effectiveness in the treatment of POP, an incredibly common condition of aging women. The involvement of multiple Canadian centers increases generalizability of findings, showcases the needed elements for adoption of a new technique in common surgical practice and promises broad clinical impact both nationally and worldwide.

## Supporting information

S1 Checklist SPIRIT checklist: Recommended items to address in a clinical trial protocol and related documents*. (DOC)

S1 File Original protocol approved by ethics board. (DOCX)
