# Correction: COMET (Composite Outcomes of Mesh vs suture Techniques for prolapse repair)- Protocol for a single blind randomized controlled multicenter trial testing surgical innovation in female pelvic surgery

**DOI:** 10.1371/journal.pone.0331750

**Published:** 2025-09-04

**Authors:** Lina Roa, Maryse Larouche, Momoe Hyakutake, Erin A. Brennand, Ola Malabarey, Nicole Koenig, Terry Lee, Joel Singer, Wei Zhang, Lori A. Brotto, Roxana Geoffrion

The word “single” is incorrect in the title. The correct title is: COMET (Composite Outcomes of Mesh vs suture Techniques for prolapse repair)- Protocol for a double blind randomized controlled multicenter trial testing surgical innovation in female pelvic surgery. The correct citation is: Roa L, Larouche M, Hyakutake M, Brennand EA, Malabarey O, Koenig N, et al. (2024) COMET (Composite Outcomes of Mesh vs suture Techniques for prolapse repair)- Protocol for a double blind randomized controlled multicenter trial testing surgical innovation in female pelvic surgery. PLoS ONE 19(10): e0308926. https://doi.org/10.1371/journal.pone.0308926.
