# Supplementary material for: COMET (Composite Outcomes of Mesh vs suture Techniques for prolapse repair)- Protocol for a single blind randomized controlled multicenter trial testing surgical innovation in female pelvic surgery
Source: PLoS One. 2024 Oct 24;19(10):e0308926. doi: 10.1371/journal.pone.0308926 (PMC11500844; doi:10.1371/journal.pone.0308926)
Supplement: S1 Checklist — (DOC) [file pone.0308926.s001.doc]

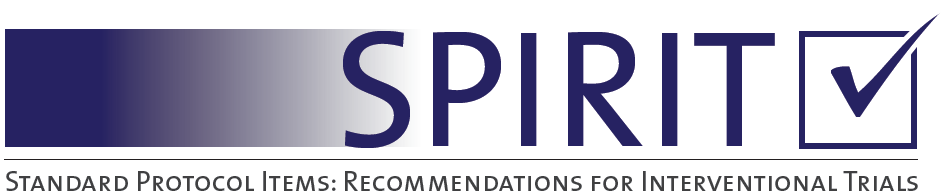


SPIRIT Checklist: Recommended items to address in a clinical trial protocol and related documents*

| Section/item | ItemNo | Description |
| --- | --- | --- |
| **Administrative information** | | |
| Title | 1 | Descriptive title identifying the study design, population, interventions, and, if applicable, trial acronym  Line 1 and 2 |
| Trial registration | 2a | Trial identifier and registry name. If not yet registered, name of intended registry  Line 64 and 65 |
|  | 2b | All items from the World Health Organization Trial Registration Data Set |
| Protocol version | 3 | Date and version identifier  This is the latest version-July 2023 |
| Funding | 4 | Sources and types of financial, material, and other support  Line 30 |
| Roles and responsibilities | 5a | Names, affiliations, and roles of protocol contributors  Line 6-29 |
|  | 5b | Name and contact information for the trial sponsor  Nicole Koenig  Email: NKoenig@providencehealth.bc.ca |
|  | 5c | Role of study sponsor and funders, if any, in study design; collection, management, analysis, and interpretation of data; writing of the report; and the decision to submit the report for publication, including whether they will have ultimate authority over any of these activities  Line 30-32 |
|  | 5d | Composition, roles, and responsibilities of the coordinating centre, steering committee, endpoint adjudication committee, data management team, and other individuals or groups overseeing the trial, if applicable (see Item 21a for data monitoring committee) |
| Introduction |  | Drs. Geoffrion, Cundiff, Larouche, Singer and research coordinator Nicole Koenig will ensure safety and scientific integrity of the study by monitoring the study data and making executive decisions on modification of the trial as needed. Meetings will be scheduled every 3 months via teleconference and more meetings will be organized on an ad hoc basis. |
| Background and rationale | 6a | Description of research question and justification for undertaking the trial, including summary of relevant studies (published and unpublished) examining benefits and harms for each intervention  Line 75 -110 |
|  | 6b | Explanation for choice of comparators  Line 79-88 |
| Objectives | 7 | Specific objectives or hypotheses  Line 113-119 |
| Trial design | 8 | Description of trial design including type of trial (eg, parallel group, crossover, factorial, single group), allocation ratio, and framework (eg, superiority, equivalence, noninferiority, exploratory)  Line 113 |
| Methods: Participants, interventions, and outcomes | | |
| Study setting | 9 | Description of study settings (eg, community clinic, academic hospital) and list of countries where data will be collected. Reference to where list of study sites can be obtained  Line 127-128 |
| Eligibility criteria | 10 | Inclusion and exclusion criteria for participants. If applicable, eligibility criteria for study centres and individuals who will perform the interventions (eg, surgeons, psychotherapists)  Line 131-149 |
| Interventions | 11a | Interventions for each group with sufficient detail to allow replication, including how and when they will be administered  Line 185-191 |
|  | 11b | Criteria for discontinuing or modifying allocated interventions for a given trial participant (eg, drug dose change in response to harms, participant request, or improving/worsening disease)  Once the allocated intervention (type of surgery) is assigned to a patient and the surgery is performed, it is not possible to modify the intervention |
|  | 11c | Strategies to improve adherence to intervention protocols, and any procedures for monitoring adherence (eg, drug tablet return, laboratory tests)  No adherence to intervention is necessary as the intervention is a surgical procedure |
|  | 11d | Relevant concomitant care and interventions that are permitted or prohibited during the trial  None. Patients are encouraged to follow routine post-operative care recommendations. |
| Outcomes | 12 | Primary, secondary, and other outcomes, including the specific measurement variable (eg, systolic blood pressure), analysis metric (eg, change from baseline, final value, time to event), method of aggregation (eg, median, proportion), and time point for each outcome. Explanation of the clinical relevance of chosen efficacy and harm outcomes is strongly recommended  Line 193-241 |
| Participant timeline | 13 | Time schedule of enrolment, interventions (including any run-ins and washouts), assessments, and visits for participants. A schematic diagram is highly recommended (see Figure)  Figure 1 |
| Sample size | 14 | Estimated number of participants needed to achieve study objectives and how it was determined, including clinical and statistical assumptions supporting any sample size calculations  Line 243-255 |
| Recruitment | 15 | Strategies for achieving adequate participant enrolment to reach target sample size  Line 152-163 |
| **Methods: Assignment of interventions (for controlled trials)** | | |
| Allocation: |  |  |
| Sequence generation | 16a | Method of generating the allocation sequence (eg, computer-generated random numbers), and list of any factors for stratification. To reduce predictability of a random sequence, details of any planned restriction (eg, blocking) should be provided in a separate document that is unavailable to those who enrol participants or assign interventions  Line 166-168 |
| Allocation concealment mechanism | 16b | Mechanism of implementing the allocation sequence (eg, central telephone; sequentially numbered, opaque, sealed envelopes), describing any steps to conceal the sequence until interventions are assigned  Line 166-168 |
| Implementation | 16c | Who will generate the allocation sequence, who will enrol participants, and who will assign participants to interventions?  Line 166-174 |
| Blinding (masking) | 17a | Who will be blinded after assignment to interventions (eg, trial participants, care providers, outcome assessors, data analysts), and how  Line 172-174 |
|  | 17b | If blinded, circumstances under which unblinding is permissible, and procedure for revealing a participant’s allocated intervention during the trial  If the patient withdraws from the trial and wishes to know which group it was allocated to |
| **Methods: Data collection, management, and analysis** | | |
| Data collection methods | 18a | Plans for assessment and collection of outcome, baseline, and other trial data, including any related processes to promote data quality (eg, duplicate measurements, training of assessors) and a description of study instruments (eg, questionnaires, laboratory tests) along with their reliability and validity, if known. Reference to where data collection forms can be found, if not in the protocol  Line 207 to 249 |
|  | 18b | Plans to promote participant retention and complete follow-up, including list of any outcome data to be collected for participants who discontinue or deviate from intervention protocols.  If patients are lost to follow up, no further data is collected. If there are any deviations to the surgical procedure this is noted. |
| Data management | 19 | Plans for data entry, coding, security, and storage, including any related processes to promote data quality (eg, double data entry; range checks for data values). Reference to where details of data management procedures can be found, if not in the protocol  Line 278-286 |
| Statistical methods | 20a | Statistical methods for analysing primary and secondary outcomes. Reference to where other details of the statistical analysis plan can be found, if not in the protocol  Line 257 to 277 |
|  | 20b | Methods for any additional analyses (eg, subgroup and adjusted analyses)  Line 272-277 |
|  | 20c | Definition of analysis population relating to protocol non-adherence (eg, as randomised analysis), and any statistical methods to handle missing data (eg, multiple imputation)  Line 259 to 269 |
| **Methods: Monitoring** | | |
| Data monitoring | 21a | Composition of data monitoring committee (DMC); summary of its role and reporting structure; statement of whether it is independent from the sponsor and competing interests; and reference to where further details about its charter can be found, if not in the protocol. Alternatively, an explanation of why a DMC is not needed  Line 293-297  An independent committee consisting of the Data safety and monitoring board (DSMB) who will monitor this trial according to international standards established by the DAMOCLES study group. We will adapt a DSMB charter that has been used by other  trials at our institution. The independent DSMB will be comprised of a clinician and a biostatistician. CIHR-funded Dr Paul Yong (clinical trial investigator) will be the chair of the DSMB. Both are at arm’s length from the project. Before joining the committee, the individuals will be able to review and agree with the trial protocol, with their responsibilities related to participation in an RCT data monitoring committee and with the range of recommendations available as part of this committee.  Each collaborator at each site is responsible for reporting serious or unexpected adverse events in an expedited manner to the main study site (Vancouver, BC).  Every six months, or upon request of the DSMB, the data coordinating centre in Vancouver will summarize all adverse events (serious, unexpected and expected) by treatment group in a report.  to the DSMB and the PI. In addition, the report will contain recruitment and drop-out rates. |
|  | 21b | Description of any interim analyses and stopping guidelines, including who will have access to these interim results and make the final decision to terminate the trial  Data safety and monitoring board (DSMB) and Steering Committee meetings will be scheduled at a minimum frequency of 6 months or more frequently in case of unexpected occurrences related to the trial. Adverse events will be reviewed at each meeting. Committee recommendations on trial cessation, trial modifications or trial continuation will be carefully reviewed and applied by the PI, co-investigators, and collaborators. At cessation of the trial, the data monitoring committee will ensure that adequate reporting and knowledge translation occurs. |
| Harms | 22 | Plans for collecting, assessing, reporting, and managing solicited and spontaneously reported adverse events and other unintended effects of trial interventions or trial conduct  Adverse events will be assessed and managed clinically by each surgeon in a similar fashion as any adverse event from surgery would be managed. Any adverse events will be reported tto the Data safety and monitoring board (DSMB) |
| Auditing | 23 | Frequency and procedures for auditing trial conduct, if any, and whether the process will be independent from investigators and the sponsor  None |
| Ethics and dissemination | | |
| Research ethics approval | 24 | Plans for seeking research ethics committee/institutional review board (REC/IRB) approval  Line 289-292 |
| Protocol amendments | 25 | Plans for communicating important protocol modifications (eg, changes to eligibility criteria, outcomes, analyses) to relevant parties (eg, investigators, REC/IRBs, trial participants, trial registries, journals, regulators)  The PI and research coordinator will oversee the communication of any important modifications to relevant parties |
| Consent or assent | 26a | Who will obtain informed consent or assent from potential trial participants or authorised surrogates, and how (see Item 32)  Line 153-156 |
|  | 26b | Additional consent provisions for collection and use of participant data and biological specimens in ancillary studies, if applicable  Not applicable |
| Confidentiality | 27 | How personal information about potential and enrolled participants will be collected, shared, and maintained in order to protect confidentiality before, during, and after the trial  Line 279-286 |
| Declaration of interests | 28 | Financial and other competing interests for principal investigators for the overall trial and each study site  None |
| Access to data | 29 | Statement of who will have access to the final trial dataset, and disclosure of contractual agreements that limit such access for investigators  The statistician as well as the research lead at each site will have access to the finalized dataset. |
| Ancillary and post-trial care | 30 | Provisions, if any, for ancillary and post-trial care, and for compensation to those who suffer harm from trial participation  Participants will receive routine post-op care in the same fashion as any other patient undergoing this surgical procedure. |
| Dissemination policy | 31a | Plans for investigators and sponsor to communicate trial results to participants, healthcare professionals, the public, and other relevant groups (eg, via publication, reporting in results databases, or other data sharing arrangements), including any publication restrictions  Knowledge translation strategies in this trial will include informing the general public about pelvic organ prolapse, its treatment and the effect of surgery on improving quality of life.  The results will be presented at national and international a national meeting as well as nation and international professional meetings. The findings will be published in academic journals and shared via social media. |
|  | 31b | Authorship eligibility guidelines and any intended use of professional writers  Professional writers will not be used. All authors will meet the ICMJE authorship criteria. |
|  | 31c | Plans, if any, for granting public access to the full protocol, participant-level dataset, and statistical code  Currently, we have no plans to share participant-level data sets or statistical code. The protocol will be published in an academic journal |
| Appendices |  |  |
| Informed consent materials | 32 | Model consent form and other related documentation given to participants and authorised surrogates  See Appendix #3 |
| Biological specimens | 33 | Plans for collection, laboratory evaluation, and storage of biological specimens for genetic or molecular analysis in the current trial and for future use in ancillary studies, if applicable  Not applicable |

*It is strongly recommended that this checklist be read in conjunction with the SPIRIT 2013 Explanation & Elaboration for important clarification on the items. Amendments to the protocol should be tracked and dated. The SPIRIT checklist is copyrighted by the SPIRIT Group under the Creative Commons “[Attribution-NonCommercial-NoDerivs 3.0 Unported](http://www.creativecommons.org/licenses/by-nc-nd/3.0/)” license.
