# Supplementary material for: COMET (Composite Outcomes of Mesh vs suture Techniques for prolapse repair)- Protocol for a single blind randomized controlled multicenter trial testing surgical innovation in female pelvic surgery
Source: PLoS One. 2024 Oct 24;19(10):e0308926. doi: 10.1371/journal.pone.0308926 (PMC11500844; doi:10.1371/journal.pone.0308926)
Supplement: S1 File — (DOCX) [file pone.0308926.s002.docx]

**Brief Summary**

This is a Canadian, multi-centre, double-blind randomized controlled trial of an **innovative** vaginal surgery technique for correction of pelvic organ prolapse (POP) in women. Vaginal surgery is preferred as minimally invasive, however we do not know if materials such as synthetic polypropylene mesh improve success, durability and cost-effectiveness. Our principal goal is to compare our experimental bilateral sacrospinous vaginal vault fixation with **synthetic mesh arms (BSSVF-M)** to the current standard of sacrospinous ligament suspension with **synthetic sutures (SSLS)** over a timeline of 2 years. Patients and evaluators will be blind to technique.

**Trial Registration**

ClinicalTrials.gov

**Funding** CIHR 2016-2021

**Roles and Responsibilities**

*Principal Investigator*: Roxana Geoffrion MD (UBC)

*Co-Investigators*: Lori Brotto, PhD (UBC); Joel Singer, PhD (UBC Centre for Health Evaluation and Outcome Sciences); Wei Zhang, PhD (UBC Centre for Health Evaluation and Outcome Sciences).

*Collaborators*:

- Surgeons (and knowledge users): Geoffrey Cundiff MD (Vancouver), Momoe Hyakutake MD (Edmonton), Maryse Larouche MD and Jens Erik Walter MD (Montreal),) Chelsea Elwood MD (Vancouver) Fariba Mohtashami MD (Langley) Peter Kruger MD (Edmonton), May Sanaee MD (Edmonton), Erin Kelly MD (Edmonton) Ola Malabarey MD (Hamilton) Dr. Annick Poirier (Edmonton) Erin Brennand (Calgary) Colin Birch (Calgary) Shunana Kim-Fine (Calgary)
- Biostatistician: Terry Lee, PhD (UBC Centre for Health Evaluation and Outcome Sciences).

*Coordinating centre*: Vancouver

**Introduction**

**Background and Rationale**

The pelvic floor comprises muscles, nerves and a fascial network with dynamic, elastic ligaments of support. Pelvic organ prolapse (POP) occurs when the capacity to accommodate pressure and stretch is exceeded and increases with childbirth and aging.^1^ One in five women need surgery for POP.^2^ POP suspension performed vaginally is among the most important ideas in gynecology in the past few decades, however we do not know which technique is best. The nominated principal applicant (NPA) has developed a novel surgery: bilateral sacrospinous vaginal vault fixation with synthetic mesh arms (BSSVF-M), which may be more successful, durable and cost-effective than standard sacrospinous ligament suspension with sutures (SSLS) and may thus become the preferred vaginal POP surgery both nationally and internationally. A Cochrane review on surgical management of POP concluded that *“adequately powered RCTs with blinding of assessors are urgently needed […] they particularly need to include women's perceptions.”*^3^ The OPTIMAL trial, a large RCT comparing 2 POP surgeries, established a stringent definition of success using a composite outcome of objectively measured POP, subjective improvement and the need for another treatment for POP recurrence.^4^

Identifying the best way to surgically suspend the vagina for POP can make the difference between a woman who can enjoy a desired level of physical activity at work or play, empty her bowel and bladder without concerns, engage in a normal sexual life and a woman who can’t perform these basic activities without discomfort, pain, worry and shame.^5-8^ The vaginal approach for pelvic surgery offers less pain, a shorter hospital stay, a faster recovery and no external incisions.^3^ However, **what is the safest, most durable, most cost-effective vaginal POP surgery?** Prior operations have produced unacceptable complications or a need for reoperation for POP recurrence.^9^ Further suffering for women and unacceptable healthcare costs ensued. Because seniors (over age 65) are the fastest growing age group in Canada, the CIHR states research into aging should be prioritized.^10^ By 2031, Statistics Canada estimates the number of senior women to reach 5.1 million or 24% of the total female population.^11^ Women have longer life expectancy than men.^11^ The burden of POP increases with age; 1 of 2 women over 80 experience pelvic floor dysfunction.^1^ For some aging women, vaginal surgery is the only option because of comorbidities precluding more invasive or lengthier open abdominal surgeries.^9^ Currently, there is true clinical equipoise between BSSVF-M and SSLS. **Our novel mesh insertion technique, BSSVF-M, offers the advantages of small size, deep, tension-free and tailored to individual anatomy**. This ensures midline restoration of the vaginal axis^12^ and maintains flexibility for normal bladder, bowel and sexual function.^13^ Tension-free insertion of BSSVF-M is a key difference with SSLS, which pulls the vagina tight against ligaments, causing pain or the return of POP after SSLS. Surgical innovation is important but adoption of new procedures needs reliable evidence acquired through a rigorous **I**nnovation, **D**evelopment, **E**xploration, **A**ssessment, **L**ong-term study (IDEAL) paradigm.^14^

We have been studying BSSVF-M over the past 9 years. Initial development included a magnetic resonance imaging study establishing restoration of pelvic anatomy comparable to normal.^12^ An exploratory prospective cohort study followed, showing BSSVF-M to be safe, easily taught to other surgeons and successful in 77% of women at one year, with the OPTIMAL composite outcome.^13^ This is superior to the reported 60% success of the SSLS^13^ and informed our power calculation for the proposed RCT. An informal survey of 50 Western Society for Pelvic Medicine surgeons indicated that an average of 14% increase in composite outcome success would be sufficient to adopt a new vaginal suspension technique. We are now at the IDEAL^14^ assessment stage, where our novel technique warrants an RCT before widespread adoption. The results of this trial will directly inform surgeons whether mesh should be used for vaginal repair of POP and how to counsel patients. This trial includes knowledge translation for women to understand pelvic floor symptoms, related condition-specific quality of life, sexual health and body image after two vaginal surgery procedures. A rigorous cost effectiveness analysis will inform different stakeholders and policy makers regarding cost effectiveness.

**Primary Objective**

To compare BSSVF-M *vs.* SSLS via the OPTIMAL composite outcome measure at 2 years.

**Secondary Objectives**

To determine condition-specific urinary, bowel and POP symptoms, quality of life, new onset pelvic pain, gender-specific body image, sexuality, global improvement, adverse events, reoperations and health utility up to 2 years post BSSVF-M *vs.* SSLS. Validated questionnaires and adverse event schemes will be used. A Markov model will estimate 10-year health benefits and costs of surgery.

**Methods (fig 1)**

**Study Setting**

Tertiary care urogynecology centres in Vancouver, Edmonton, Montreal, Hamilton

**Eligibility**

Inclusion Criteria

- Women over 19 years of age
- Able to read and write in English, or alternatively have someone to translate for them
- Able to follow up with clinic visits for up to two years after surgery
- Diagnosed with bothersome pelvic organ prolapse including at the top of the vagina

Exclusion Criteria

- Women who wish to conserve the uterus
- Prior pelvic radiation
- Prior vaginal mesh surgery for prolapse
- Prior vaginal mesh exposure
- Presence of vaginal pain from pelvic floor muscle spasm documented at baseline visit
- Plan for synthetic vaginal mesh insertion for prolapse, at the same time, at locations other than the top of the vagina (NOTE: concurrent need for synthetic anti-incontinence sling is NOT an exclusion)
- Immune compromise; chronic steroid use
- Current smoker
- Currently pregnant or breastfeeding
- Presence of pain syndromes likely to cause a heightened sensitivity to pelvic pain (example: fibromyalgia or painful bladder syndrome)

**Interventions (fig 2):**

Patients > age 19, symptomatic POP (stage 1-4^15^) randomized to BSSVF-M or SSLS. Both surgeries are performed through the same vaginal incisions. BSSVF-M uses bilateral synthetic polypropylene mesh arms for support of the vaginal wall and SSLS uses two synthetic sutures attached uni- or bilaterally.^3,16^ As both are performed through the same incision, women will not know their treatment. Group assignment will remain blinded through trial end. Surgeons will receive group allocation immediately prior to surgery. To diminish bias, research personnel conducting pelvic exams to evaluate the primary outcome will be blind to the type of procedure.

**Outcome measures**

Primary, at 2 yrs

Composite outcome of 3 objective signs and 1 subjective symptom of POP (yes/no answers). The definition of surgical success, used in the OPTIMAL trial^4^ is the absence of all of the following: (1) objectively recorded, via POP quantification (POP-Q)^15^, recurrent POP of the top of the vagina beyond the upper third of the vaginal canal; (2) objectively recorded, via POPQ, recurrent POP of the anterior or posterior vaginal walls beyond the hymenal ring (vaginal entrance); (3) vaginal bulge symptoms reported by the patient, as indicated by an affirmative response to either: *“Do you usually have a bulge or something falling out that you can see or feel in the vaginal area?”* and any response other than *“not at all”* to the question *“How much does that bother you?”* (4) re-treatment for prolapse by either surgery or pessary (conservative treatment with insertion of a silicone ring in the vagina for support). The POPQ is a validated quantification system with adequate interrater reliability which measures, in centimeters, the extent of vaginal wall descent with respect to a reference point at the hymenal ring.^15^

Secondary, at 6 weeks, 6, 12 and 24 months

Change in maximal POP of vaginal compartments via POPQ, obtained by research personnel (nurse or clinical fellow) trained in POPQ and blinded to group allocation. POP will be quantified and compared to baseline. Numerical questionnaire scores will be compared between the two groups from baseline to 6 weeks, 12 and 24 months postoperatively. Change in questionnaire scores between the 2 groups from baseline to 6 weeks, 12 and 24 months postoperatively: Pelvic Floor Distress Inventory (PFDI-20) and the Pelvic Floor Impact Questionnaire (PFIQ-7)^17^; the short form McGill pain questionnaire^18^; a validated gender-specific body image scale specifically for POP.^8^ Change in questionnaire scores between the 2 groups from baseline to 12 and 24 months: Pelvic organ prolapse/ Incontinence Sexual Questionnaire, IUGA-Revised (PISQ-IR)^19^. Difference between global impression of improvement (Likert Scale) of the 2 groups at 6 weeks, 12 and 24 months postoperatively. Difference between the 2 groups in length of surgery, postoperative hospital stay, complications such as pain or mesh exposure, reoperations and other unexpected adverse events categorized using the Clavien Dindo validated classification system for surgical complications.^20^ Difference in health utilities measured via the EuroQol instrument (EQ-5D)^21,22^ and in direct healthcare utilization and costs collected by the healthcare service utilization questionnaire^23^ at baseline, 6 weeks, 6, 12 and 24 months postoperatively.

Health economic evaluation

A cost-effectiveness analysis (CEA) of BSSVF-M *vs*. SSLS will be conducted alongside the 2-year trial from the healthcare system perspective. The incremental cost-effectiveness ratio (ICER) during the 2-year study period will be calculated by dividing the difference in 2-year costs by the difference in the Quality Adjusted Life Years (QALYs). Bootstrap method will estimate the uncertainty surrounding ICER. In addition, an alternative CEA will be carried out over 10 years after commencement of surgical treatment by a Markov model. Published Markov models^24,25^ will be adapted to simulate transition between different health states postoperatively and to extrapolate within-trial results over 10 years. Specifically, the potential health states include repaired POP without late/post-operative complications, repaired POP with minor late complications, and repaired POP with major late complications requiring revision surgery. All costs and QALYs will be discounted at a rate of 3% per year. The ICER will be estimated. Probabilistic sensitivity analysis with 1000 Monte-Carlo simulations will be employed to assess the impact of model parameters with greater uncertainty and calculate the 95% credible interval of the ICER.

**Recruitment/Timeline (fig 3)**

We anticipate recruiting over 2.5 yrs (fall 2016-spring 2019). Total timeline for the trial is 5 yrs, to ensure follow-up of all recruited women for 2 yrs postop. The main centre in Vancouver (2 surgeons) is committed to recruiting 1-2 patients per week or 130 total. Edmonton (1 surgeon), Calgary (1 surgeon) and Montreal (2 surgeons) would recruit 76 patients each. Based on prior experience with the pilot study of BSSVF-M and OPTIMAL, we anticipate approximately 90% of invited women will volunteer and 5% of those will change their mind about participation at baseline. ^13^ Given the NPA performs 4 to 8 vaginal surgeries for POP per month, we anticipate no recruitment challenges. Based on surgical volumes and commitment to research, the other sites should also reach targets. All surgeons are experts at vaginal surgery for POP (including SSLS) and all except one have performed BSSVF-M. They do not currently offer BSSVF-M outside research studies. According to national credentialing organizations^9^ and our surgical survey, surgeons require at least 5 and on average 8 procedures to become familiar with a new vaginal POP technique. Assistance with 8 cases will be provided to the surgeon untrained to perform BSSVF-M. In addition, video instruction will be provided to standardize procedure and materials.

**Challenges/Mitigation**

Compliance problems

Compliance with follow-up may be an issue due to the follow up for 2 years. Patients will be screened for future compliance at recruitment and the coordinator at each site will follow each patient quarterly over the 2 years, asking for updated contact information at each time point.

Loss to follow-up

We anticipate similarities in loss to follow-up (15%) to OPTIMAL. To mitigate loss to follow-up, we will reimburse patient parking and traveling costs with a small stipend.

Unblinding of participants

In the event of complications arising from either surgery, patients or assessors may become aware of the group assignment. For example, if a patient develops exposure of the vaginal mesh, the pelvic examination will reveal this at various time points and surgery may be needed to remove the mesh exposed in the vagina. This may introduce bias in assessments at various time points, however it will likely not affect the primary composite outcome measure at 2 years. Based on our prior experience with BSSVF-M, we anticipate unblinding complications to be less than 5% of the total sample. The primary surgeon who deals with complications and need for reoperation is not involved in pelvic examination assessments. Most complications will be corrected by 2 years, so the blinded assessor at 2 years will be unable to identify group assignment.

**Sample size**

179 women/group (358 total). Our Western Canadian survey of 50 surgeons indicated the smallest clinically relevant difference to change surgical practice would be an absolute change of 14%. The proportion of success in the control SSLS group was 60.5% in the OPTIMAL trial^4^ and the success rate with BSSVF-M was 77% in our one-year pilot study.^13^ Our pilot study was small and designed to inform a power calculation for an RCT; it did not study long-term results, cost effectiveness or KT strategies. Studies of mesh vs sutures in surgery for urinary incontinence indicate that mesh is more successful, durable and cost-effective than sutures,^26-29^ therefore we expect similar results for POP, with sustained durability of mesh over time. Our primary analytic strategy, following the example of the OPTIMAL trial, will include only patients who come for 2-yr follow-up or who were failures on the last follow-up. Patients who do not come to their 2-yr follow-up but who were failures at last follow-up will be counted as failures. Sample size was determined based on a two-sample test of independent proportions, using two-sided ∝=0.05, anticipated successful outcome rates 60 and 75%, power=80% and yielded 152 patients/group. To account for an estimated 15% loss to follow-up (patients who never had a follow-up visit or who were deemed successful on their last follow-up), similar to the OPTIMAL trial, our sample size was increased to 179 per group.

**Allocation**

Random allocation is via a web-based allocation system. A statistician (Terry Lee, CHEOS) will generate a list of random allocations, stratified by surgeon and using randomly sized permuted blocks to prevent bias in guessing the next treatment allocation. Although all surgeons will be skilled in both techniques, differences in experience may impact outcomes. By stratifying for surgeon, we ensure similar numbers of allocations to each treatment in each hospital.

Patients will be enrolled by research personnel at each participating centre.

The site coordinator will randomize the patient prior to surgery using the web-based allocation system.

Patients will be blinded to the type of procedure received until 2 years after surgery.

Research personnel evaluating outcomes at each in person visit will be blinded to the type of procedure received by participants.

**Ethics and dissemination**

This protocol and the informed consent forms will be reviewed and approved by the Institutional Review Boards (IRBs) at each participating centre with respect to scientific content and human subject regulations. The participant education and recruitment materials (and other requested documents as necessary) will also be reviewed and approved by the ethical review bodies at each participating centre. Subsequent to initial review and approval, the responsible IRBs will review the protocol at least annually. Each collaborator will make safety and progress reports to the IRBs at least annually and within 3 months of study termination. Any modifications to the protocol will require a formal amendment with each IRB.

Trained research personnel will introduce the trial to patients who will be shown educational materials regarding pelvic organ prolapse and the trial interventions. Patients will have an informed discussion with the participating personnel who will obtain written consent for participation. Patients will receive information sheets and a copy of the consent form.

All trial-related information will be stored securely at the coordinating centre in Vancouver. All participant-related information will be stored in locked file cabinets in areas with limited access. All records that contain names or other personal identifiers will be stored separately from study records identified by code number. All local databases will be secured with password-protected access systems. Participants’ study information will not be released outside of the study without the written permission of the participant, except as necessary for monitoring by government and regulatory authorities. To ensure confidentiality, data dispersed to trial team members will be blinded of any identifying participant information.

**References**

1. Nygaard I, Barber MD, Burgio KL, Kenton K, Meikle S, Schaffer J, et al. Prevalence of symptomatic pelvic floor disorders in US women. JAMA 2008;300:1311-16
2. Smith FJ, Holman CDJ, Moorin RE et al. Lifetime risk of undergoing surgery for pelvic organ prolapse. Obstet Gynecol 2010;116:1096-1100

Maher C, Feiner B, Baessler K, Schmid C. Surgical management of pelvic organ prolapse in women. Cochrane Database Syst Rev. 2013:30;4:CD004014.

Barber MD, Brubaker L, Burgio KL et al. Comparison of 2 transvaginal surgical approaches and perioperative behavioural therapy for apical vaginal prolapse: the OPTIMAL randomized trial. JAMA 2014;311:1023-34

Kendig H, Browning CJ, Thomas SA, Wells Y. Health, lifestyle and gender influences on aging well: an Australian longitudinal analysis to guide health promotion. Front Public Health. 2014;2:70

Strickland R. Reasons for not seeking care for urinary incontinence in older community-dwelling women: A contemporary review. Urol Nurs. 2014;34:63-8

Lowder JL, Ghetti C, Nikolajski C, Oliphant SS, Zyczynski H. Body image perceptions in women with pelvic organ prolapse: a qualitative study. Am J Obstet Gynecol 2011;204:441.e1-5

Lowder JL, Ghetti C, Oliphant SS, Skoczlas LC, Swift S, Switzer GE. Body image in the pelvic organ prolapse questionnaire: development and validation. Am J Obstet Gynecol 2014;211:174.e1-9

1. Unger CA, Barber MD. Vaginal mesh in pelvic reconstructive surgery: Controversies, current use and complications. Clin Obstet Gynecol. 2015;58:740-53
2. Government of Canada. CIHR Institute of Aging. <http://www.cihr-irsc.gc.ca/e/8643.html> accessed February 2016

Statistics Canada. Women in Canada: A gender-based statistical report. <http://www.statcan.gc.ca/pub/89-503-x/89-503-x2010001-eng.htm> accessed February 2016

Nicolau-Toulouse V, Tiwari P, Lee T, Cundiff GW, Geoffrion R. Does bilateral sacrospinous fixation with synthetic mesh recreate nulliparous pelvic anatomy? An MRI evaluation. Female Pelvic Med Reconstr Surg. 2014;20(4):222-7

Geoffrion R, Hyakutake MT, Koenig NA, Lee T, Cundiff GW. Bilateral sacrospinous vault fixation with tailored synthetic mesh arms: Clinical outcomes at one year. J Obstet Gynaecol Can 2015;37(2):129-37

McCulloch P, Altman, DG, Campbell WB, et al. No surgical innovation without evaluation: the IDEAL recommendations. Lancet 2009;374:1105-12

[Bump RC](http://www.ncbi.nlm.nih.gov/pubmed/?term=Bump%20RC%5BAuthor%5D&cauthor=true&cauthor_uid=8694033), [Mattiasson A](http://www.ncbi.nlm.nih.gov/pubmed/?term=Mattiasson%20A%5BAuthor%5D&cauthor=true&cauthor_uid=8694033), [Bø K](http://www.ncbi.nlm.nih.gov/pubmed/?term=B%C3%B8%20K%5BAuthor%5D&cauthor=true&cauthor_uid=8694033), [Brubaker LP](http://www.ncbi.nlm.nih.gov/pubmed/?term=Brubaker%20LP%5BAuthor%5D&cauthor=true&cauthor_uid=8694033), [DeLancey JO](http://www.ncbi.nlm.nih.gov/pubmed/?term=DeLancey%20JO%5BAuthor%5D&cauthor=true&cauthor_uid=8694033), [Klarskov P](http://www.ncbi.nlm.nih.gov/pubmed/?term=Klarskov%20P%5BAuthor%5D&cauthor=true&cauthor_uid=8694033), [Shull BL](http://www.ncbi.nlm.nih.gov/pubmed/?term=Shull%20BL%5BAuthor%5D&cauthor=true&cauthor_uid=8694033), [Smith AR](http://www.ncbi.nlm.nih.gov/pubmed/?term=Smith%20AR%5BAuthor%5D&cauthor=true&cauthor_uid=8694033). The standardization of terminology of female pelvic organ prolapse and pelvic floor dysfunction. Am J Obstet Gynecol. 1996;175(1):10-7

Richter K, Albrich W. Long-term results following fixation of vagina on the sacrospinal ligament by the vaginal route. Am J Obstet Gynecol 1981;141:811–6

1. Barber MD, Walters MD, Bump RC. Short forms of two condition-specific quality-of-life questionnaires for women with pelvic floor disorders (PFDI-20 and PFIQ-7). Am J Obstet Gynecol 2005;193:103-13
2. Melzack R. The short-form McGill Pain Questionnaire. Pain 1987;30:190-7
3. Rogers RG, Rockwood TH, Constantine ML, et al. A new measure of sexual function in women with pelvic floor disorders (PFD): the pelvic organ prolapse/incontinence sexual questionnaire, IUGA-revised (PISQ-IR). Int Urogynecol J. 2013;24:1091-103
4. Dindo D, Demartines N, Clavien PA. Classification of surgical complications: a new proposal with evaluation in a cohort of 6336 patients and results of a survey. Ann Surg. 2004;240:205-13
5. Brooks R. EuroQol: the current state of play. Health Policy1996;37:53–72
6. EuroQol Group. EuroQol–a new facility for the measurement of health-related quality of life. Health Policy 1990;16:199–208

Rand Health. The three main interview waves of HCSUS. <http://www.rand.org/health/projects/hcsus/questionnaires.html> accessed February 2016.

1. Jacklin P, Duckett J. A decision-analytic Markov model to compare the cost-utility of anterior repair augmented with synthetic mesh compared with non-mesh repair in women with surgically treated prolapse. BJOG 2013;120(2):217-23. doi:10.1111/1471-0528.12028.
2. Hullfish KL, Trowbridge ER, Stukenborg GJ. Treatment strategies for pelvic organ prolapse: a cost-effectiveness analysis. Int Urogynecol J. 2011;22(5):507-15. doi:10.1007/s00192-011-1383-6

Novara G, Artibani W, Barber MD, Chapple CR, Costantini E, Ficarra V, Hilton P, Nilsson CG, Waltregny D. Updated systematic review and meta-analysis of the comparative data on colposuspensions, pubovaginal slings, and midurethral tapes in the surgical treatment of female stress urinary incontinence. Eur Urol. 2010 Aug;58(2):218-38

Valpas A, Ala-Nissilä S, Tomas E, Nilsson CG. TVT versus laparoscopic mesh colposuspension: 5-year follow-up results of a randomized clinical trial. Int Urogynecol J. 2014 Jul 24. [Epub ahead of print]

Laudano MA, Seklehner S, Chughtai B, Lee U, Tyagi R, Kavaler E, Te AE, Kaplan SA, Lee RK. Cost-effectiveness analysis of tension-free vaginal tape vs burch colposuspension for female stress urinary incontinence in the USA. BJU Int. 2013 Jul;112(2):E151-8.

Nilsson CG, Palva K, Aarnio R, Morcos E, Falconer C. Seventeen years' follow-up of the tension-free vaginal tape procedure for female stress urinary incontinence. Int Urogynecol J. 2013 Aug;24(8):1265-9.

1. DAMOCLES Study group, NHS Health Technology Assessment Programme. A proposed charter for clinical trial data monitoring committees: helping them to do their job well. Lancet, 2005;365(9460):711-22
2. Geoffrion R, Gebhart J, Dooley Y, Bent A, Dandolu V, Meeks R, Baker K, Tang S, Ross S, Robert M. The mind’s scalpel in surgical education: a randomized controlled trial of mental imagery. BJOG 2012;119(9):1040-8

**Figure 1: Trial Flowchart:** Describes participant assessments from baseline to 2 years postoperatively

|  |  | **ELIGIBILITY ASSESSMENT** | |  |
| --- | --- | --- | --- | --- |
|  | **Not eligible/Refuses** | **Consent** | |  |
|  |  | Baseline data, questionnaires, pelvic exam (POP Quantification) | |  |
|  |  |  |  |  |
|  |  | **Randomize** | |  |
|  |  |  |  |  |
| **BSSVF-M** | | **BASELINE GROUPS** | | **SSLS** |
| Questionnaires:  Self-reported Charlson Comorbidity Index  Pelvic Floor Distress Inventory – 20 (PFDI-20)  Pelvic Floor Impact Questionnaire – 7 (PFIQ-7)  Pelvic organ prolapse/Incontinence Sexual Questionnaire (PISQ-IR)  The short form McGill Pain Questionnaire (McGill PQ)  The Body Image in Pelvic Organ Prolapse Questionnaire (BIPOP)  EQ-5D-3L Questionnaire (within the last 6 months)  Healthcare service utilization questionnaire (within the last 6 months) | | | | |
| OR details | | **INTRAOP** | | OR details |
| 6 week POP Quantification  Clavien Dindo complication scale | | **POSTOP** | | 6 week POP Quantification  Clavien Dindo complication scale |
| Questionnaires:  PFDI-20  PFIQ-7  McGill PQ  BIPOP  Global Question of Improvement  EQ-5D-3L  Healthcare service utilization questionnaire | | | | |
| 6 month phone call | | **PHONE EVALUATION** | | 6 month phone call |
| Questionnaires:  EQ-5D-3L  Health care service utilization questionnaire | | | | |
| 12 month questionnaires  POP Quantification  Clavien Dindo complication scale | | **MID-TRIAL ASSESSMENT** | | 12 month questionnaires  POP Quantification  Clavien Dindo complication scale |
| Questionnaires:  PFDI-20  PFIQ-7  PISQ-IR  McGill PQ  BIPOP  Global Question of Improvement  EQ-5D-3L  Healthcare service utilization questionnaire | | | | |
| 24 month questionnaires  POP Quantification  Clavien Dindo complication scale | | **FINAL ASSESSMENT** | | 24 month questionnaires  POP Quantification  Clavien Dindo complication scale |
| Questionnaires:  PFDI-20  PFIQ-7  PISQ-IR  McGill PQ  BIPOP  Global Question of Improvement  EQ-5D-3L  Healthcare service utilization questionnaire | | | | |

**Figure 2:** **Vault suspension techniques:** a) **BSSVF-M**^13^ – novel technique performed with bilateral synthetic mesh arms attached to sacrospinous ligaments to replace the vagina tension-free in its normal anatomic orientation within the pelvis, can carry risks of mesh exposure and pain; b) **SSLS** - standard technique mostly performed with unilateral (but can be bilateral) synthetic sutures attached to sacrospinous ligaments, tensioned and anatomically incorrect, can carry risks of pain, detachment and prolapse recurrence.


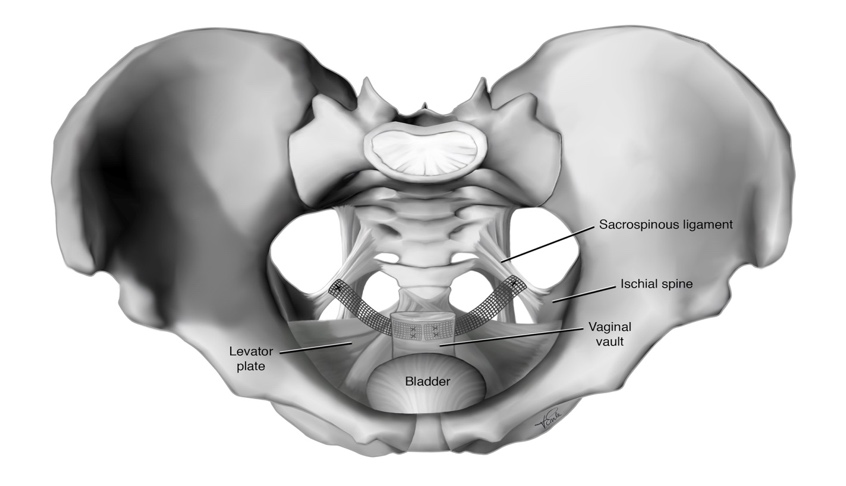

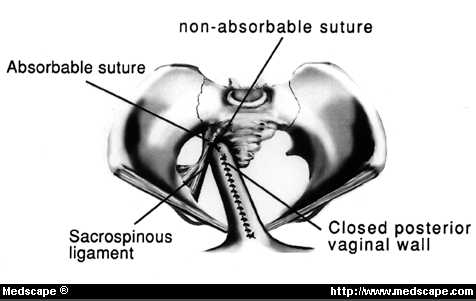


1. Bilateral Sacrospinous Vault Fixation b) Unilateral Sacrospinous Ligament

with **Mesh arms (BSSVF-M)^13^** Suspension with **synthetic sutures (SSLS)**

**Figure 3: Trial Timeline**

| **TIMELINE** | **2016/2017** | | | | **2017/2018** | | | | **2018/2019** | | | | **2019/2020** | | | | **2020/2021** | | | |
| --- | --- | --- | --- | --- | --- | --- | --- | --- | --- | --- | --- | --- | --- | --- | --- | --- | --- | --- | --- | --- |
| Activity | Q1 | Q2 | Q3 | Q4 | Q1 | Q2 | Q3 | Q4 | Q1 | Q2 | Q3 | Q4 | Q1 | Q2 | Q3 | Q4 | Q1 | Q2 | Q3 | Q4 |
| Set up and approvals |  |  |  |  |  |  |  |  |  |  |  |  |  |  |  |  |  |  |  |  |
| Recruitment |  |  |  |  |  |  |  |  |  |  |  |  |  |  |  |  |  |  |  |  |
| Enrollment visit |  |  |  |  |  |  |  |  |  |  |  |  |  |  |  |  |  |  |  |  |
| Study Surgery |  |  |  |  |  |  |  |  |  |  |  |  |  |  |  |  |  |  |  |  |
| 6 weeks visit |  |  |  |  |  |  |  |  |  |  |  |  |  |  |  |  |  |  |  |  |
| 6 months call |  |  |  |  |  |  |  |  |  |  |  |  |  |  |  |  |  |  |  |  |
| 12 months visit |  |  |  |  |  |  |  |  |  |  |  |  |  |  |  |  |  |  |  |  |
| 24 months visit |  |  |  |  |  |  |  |  |  |  |  |  |  |  |  |  |  |  |  |  |
| Data monitoring |  |  |  |  |  |  |  |  |  |  |  |  |  |  |  |  |  |  |  |  |
| Statistical Analysis |  |  |  |  |  |  |  |  |  |  |  |  |  |  |  |  |  |  |  |  |
| Health Economic Analysis |  |  |  |  |  |  |  |  |  |  |  |  |  |  |  |  |  |  |  |  |
| Knowledge Translation |  |  |  |  |  |  |  |  |  |  |  |  |  |  |  |  |  |  |  |  |
